# Pathogen Eradication in Garlic in the Phytobiome Context: Should We Aim for Complete Cleaning?

**DOI:** 10.3390/plants12244125

**Published:** 2023-12-10

**Authors:** Itay Yarmus, Dana Gelbart, Einat Shemesh-Mayer, Doron Dov Teper, Dana Ment, Adi Faigenboim, Ross Peters, Rina Kamenetsky-Goldstein

**Affiliations:** 1 Agricultural Research Organization, the Volcani Center, Risho LeZion 7505101, Israel; itay.yarmus@mail.huji.ac.il (I.Y.); danag@volcani.agri.gov.il (D.G.); shemeshe@volcani.agri.gov.il (E.S.-M.); doront@volcani.agri.gov.il (D.D.T.); danam@volcani.agri.gov.il (D.M.); adif@volcani.agri.gov.il (A.F.); rossp@volcani.agri.gov.il (R.P.); 2 The Robert H. Smith Faculty of Agriculture, Food and Environment, The Hebrew University of Jerusalem, Jerusalem 9190501, Israel

**Keywords:** *Allium sativum*, fungi, bacteria, potyvirus, carlavirus, allexivirus, cryopreservation, tissue culture

## Abstract

Global food production is challenged by plant pathogens that cause significant crop losses. Fungi, bacteria, and viruses have long threatened sustainable and profitable agriculture. The danger is even higher in vegetatively propagated horticultural crops, such as garlic. Currently, quarantine, rouging infected plants, and control of natural vectors are used as the main means of disease and pest control in garlic crops. Agricultural biotechnology, meristem-tip culture, and cryotherapy offer solutions for virus eradication and for the multiplication of ‘clean stocks’, but at the same time, impact the symbiotic and beneficial components of the garlic microbiome. Our research involves the first metatranscriptomic analysis of the microbiome of garlic bulb tissue, PCR analyses, and a biological assay of endophytes and pathogens. We have demonstrated that in vitro sanitation methods, such as shoot tip culture or cryotherapy can alter the garlic microbiome. Shoot tip culture proved ineffective in virus elimination, but reduced bacterial load and eliminated fungal infections. Conversely, cryotherapy was efficient in virus eradication but demolished other components of the garlic microbiome. Garlic plants sanitized by cryotherapy exhibited a lower survival rate, and a longer in vitro regeneration period. The question arises whether total eradication of viruses, at the expense of other microflora, is necessary, or if a partial reduction in the pathogenic load would suffice for sanitized garlic production. We explore this question from both scientific and commercial perspectives.

## 1. Introduction

Global food production is challenged by plant pathogens that cause significant crop losses [1]. Fungi, bacteria, and obligate pathogens such as viruses, viroids, or phytoplasmas have long threatened sustainable and profitable agriculture [2]. The danger is even higher in vegetatively propagated horticultural crops, such as potatoes, yam, banana, and grapes, where pathogen load accumulates from one production cycle to another. Therefore, pathogen-free stock plants are produced in quarantine programs, and healthy propagation materials are used in nurseries and in horticultural trade [3,4]. In this context, garlic (*Allium sativum* L.) is certainly one of the most challenging crops. Commercial varieties of garlic are propagated only vegetatively. After harvest, the underground bulb is stored for a few months and then separated into cloves that serve as propagules for the next production cycle. This system expedites not only contamination by field and storage pathogens but also a gradual accumulation of the pathogen load. The disease compendium of garlic includes bacteria (e.g., Pseudomonas Pectobacterium), fungi and oomycetes (Penicillium, Botrytis, Peronospora, Alternaria, Fusarium), parasitic nematodes, phytoplasmas, and viruses [5]. Most pathogens live in the plants for years and might severely damage a garlic crop. The list of pathogens is constantly growing. *Sclerotium cepivorum*, *Bacillus siamensis*, and *Streptomyces setonii* were recently detected in garlic in different sites in China [6]. In Argentina, the world’s second-largest exporter of garlic, *Penicillium viridicatum*, *P. hirsutum* and *P. allii* are the causal agents of blue mold [7]. In Egypt, isolates of *Fusarium oxysporum*, *F. proliferatum* and *F. solani* caused the highest rates of clove rot [8].

Viral infections in garlic are regarded as the most devastating [9]. Currently, the garlic virome includes at least 20 members [10] which vary in virulence and the degree of damage they cause to the crop. Garlic viruses A-to-D (GVA, GVB, GVC, and GVD), Garlic virus X (GVX), and Garlic mite-borne mosaic virus (GMbMV) belong to allexiviruses and are transmitted by eriophyid mites. Potyviruses Garlic mosaic virus (GMV), Leek yellow stripe virus (LYSV) and Onion yellow dwarf virus (OYDV) are transmitted by aphids. Carlaviruses, transmitted by aphids or other insects, include Garlic latent virus (GLV), Garlic common latent virus (GCLV) and Shallot latent virus (SLV). Iris yellow spot virus (IYSV) (Tospoviridade) is transmitted by thrips and infects both garlic and onion [11]. Potyviruses are recognized as the most dangerous pathogens, but Allexiviruses and Carlaviruses also weaken garlic plants and reduce crop productivity [12,13].

Prophylaxis (quarantine, roguing infected plants, and control of natural vectors) is the main means of pathogen restriction [14]. While in other crops, modern defense strategies of breeding for genetic resistance (e.g., transformation, gene editing or cross-protection) are under development, they are still impractical for garlic, due to the absence of reliable regeneration methods. Therefore, the main strategies to keep propagation stock clean are pathogen eradication and protected cultivation of “clean” stocks.

In the absence of sexual reproduction of garlic by seed, pathogen-free plant material of garlic might be achieved by in vitro procedures, such as meristem culture, thermotherapy, chemotherapy, or cryotherapy [15,16]. Taşkin et al. [17] reported that meristem culture could completely eradicate garlic’s virulent viruses, OYDV and LYSV, while shoot tip culture was less effective and resulted in the presence of 73% and 87% of OYDV and LYSV in treated plants, respectively. Vitrification cryo-methods have been developed for garlic gene bank collections [3,18]. Cryotherapy might eliminate plant pathogens such as viruses, phytoplasmas and bacteria by brief immersion of shoot tips in liquid nitrogen. Healthy plants are regenerated from the surviving pathogen-free young meristematic tissue. Although shoot tip regeneration rates are low after cryotherapy in comparison with traditional meristem culture, explant excision is easier and the regenerants are pathogen-free [19].

After in vitro procedures, three to five years of ex vitro propagation are required to obtain a sufficiently large population for commercial use [20,21]. Over the course of these seasons, there is the possibility of reinfection, and in any case, field production will contaminate the material again.

The question arises whether total eradication of viruses (and other microflora) is necessary, or if a partial reduction in the pathogenic load would be preferable for sanitized garlic production.

The concept of the plant as a holobiont suggests that host plants interact with the microbiotic community and that beneficial and symbiotic microorganisms play an integral role in the plant’s metabolism, nutrient uptake, stress tolerance, pathogen resistance, and other physiological processes [22,23]. Plant microbiomes include epiphytes that colonize the exterior surfaces of plants and endophytes that penetrate the epidermis and colonize intercellular and intracellular space [24]. While viruses are mostly destructive pathogens, they are also an integral part of a plant’s microbiome and may make positive contributions to balance in a plant’s life [25]. Beneficial or mutualistic symbioses of viruses with various host organisms, including bacteria, insects, fungi and plants have been discovered and reported [26]. However, a major contradiction exists between the phytobiome approach and the practical need to eradicate pathogens from agricultural crops. This issue is essential to numerous vegetatively-propagated crops, such as potatoes, cassava, citrus, cacao, and many others [27].

In this report, we employed a metatranscriptomic approach to identify endophyte taxa in garlic bulb tissues. This method has already enabled the identification of a high number of endophytes in the living plant tissues of other plants, e.g., grapes [28] and oats [29]. Using RNA sequencing (RNASeq) to record expressed transcripts provides a closer look at active members of a plant’s microbiome [30]. We have also used culture-dependent methods and identification of bacteria by 16S rRNA gene sequence analysis and PCR to detect fungi, bacteria, and viruses in the stored garlic bulbs.

Our results reveal the interface between the complex approach to the plant as a holobiont and the practical need for pathogen eradication from garlic. We estimate the garlic microbiome as a very large and variable community and show how different in vitro sanitation techniques alter the garlic microbiome.

## 2. Results

### 2.1. The Mycobiome of Garlic

**Figure 1:**
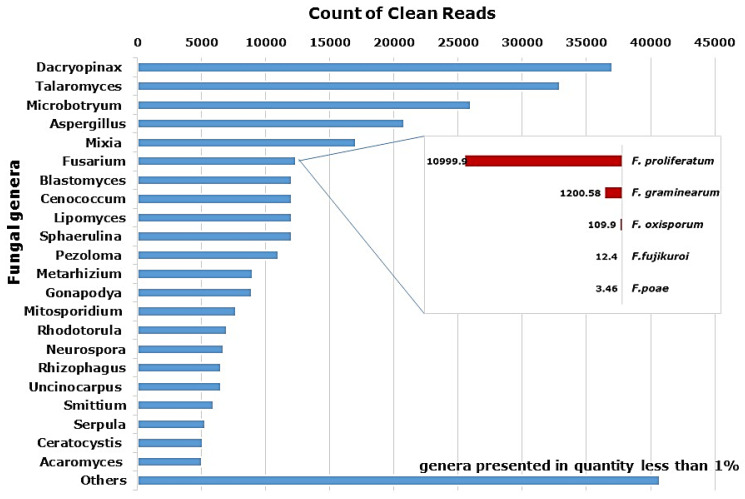
Fungal genera associated with the transcriptome of garlic ‘Shani’. Contigs homologous to fungi were isolated, merged for isoforms of very similar proteins, filtered for identification degree (above 80%), and compared with the NCBI database. Insert: account of five Fusarium species.

The full list of fungi associated with the transcriptome of garlic bulbs consisted of 96 species from 73 genera (Figure 1). Global analysis shows that garlic bulbs were enriched with saprophytic Dacryopinax (Dacrymycetaceae) (12% of read counts). The genus Aspergillus that causes storage decay in garlic is represented by 11 species. Amongst the five species of Fusarium, the species *F. proliferatum* was dominant, accounting for 89.2% out of all Fusarium reads. Other species of Fusarium found were *F. graminearum* (9.74%), *F. oxysporum* (0.89%), *F. fujikuroi* (0.1%) and *F. poae* (0.03%).

Fungal biota also included 10% Talaroromyces (formerly Penicillium), which has a large distribution in a wide range of habitats and is considered an important pathogen [31], as well as Microbotryum (8%). Other pathogenic genera were found in smaller amounts. Interestingly, the entomopathogenic fungi Metarhizium and Acaromyces were present in 3 and 2% respectively. About 13% of genera are presented in quantities of less than 1% (Figure 1).

The biological assay of exophytic and endophytic fungi in garlic cloves confirmed their presence in 87% of the samples of external segments, whereas internal segments of cloves from the warehouse showed fungal development in only 13% of the samples (Table 1). Of the cloves stored in the warehouse, about 30% of all samples developed only one fungal genus, while 13% were infected with two or more species. Mixed fungal infections originated only from the external samples.

Storage of cloves at 4 °C for eight months resulted in an increase in fungal infection. Fungi were found in 97% and 28% of the external and internal tissues, respectively, while at least 30% of samples were infected by a combination of two or even three fungal genera (Table 1).

**Figure 2:**
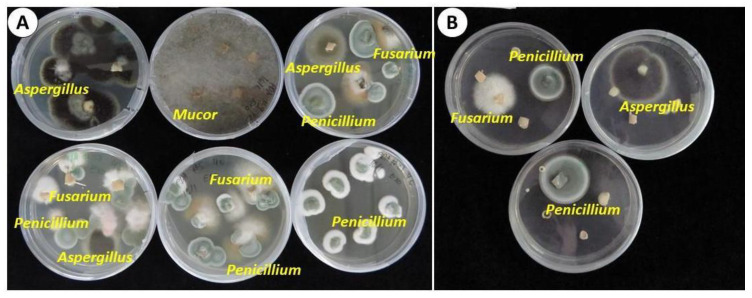
Representative samples of mycoflora of garlic ‘Shani.’ The bulbs were cultivated in an experimental field in Israel and subsequently stored for eight months in an open shed. (**A**) Epiphytic; (**B**) Endophytic. Visual identification of Aspergillus, Mucor, Penicillium, and Fusarium.

**Figure 3:**
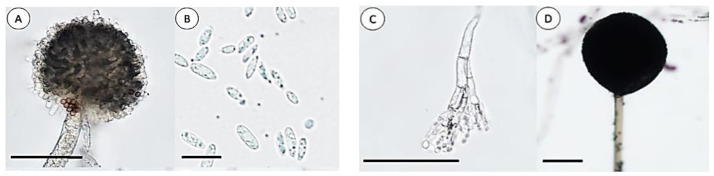
Representative images of mycelium tested on a PDA medium. Aspergillus (**A**); Fusarium (**B**); Penicillium (**C**); Mucor (**D**). Scale bar (**A**,**C**)—50 µm, (**B**,**D**)—20 µm.

Visual identification of fungi according to the structure of hyphae and conidia predominantly found in three genera: Penicillium, Aspergillus, and Fusarium (Figure 2 and Figure 3). Mucor was also found, but not commonly.

### 2.2. The Bacterial Community of Garlic

**Figure 4:**
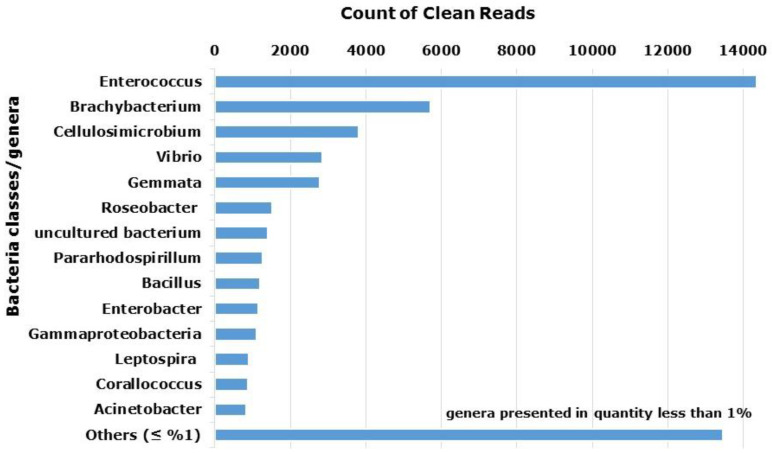
Bacterial classes/genera associated with the transcriptome of garlic ‘Shani’. Gammaproteobacteria include Vibrio, Pseudomonas, and Enterobacter. Contigs homologous to bacteria were isolated, merged for isoforms of similar proteins, filtered for identification degree (above 80%), and compared with the NCBI database.

The full list of bacteria genera found to be associated with garlic transcriptome consists of 125 species from 79 genera. Transcriptome analysis of clove-associated bacteria found an abundance of Enterococcus (27%), Brachybacterium (11%), Cellulosimicrobium (7%), and Vibrio (5%). Two genera that include pathogens of garlic (Enterobacter and Pseudomonas) were found only in small amounts. We were able to identify *P. fluorescens*, *P. savastanoi* and *P. syringae*, among others. At least 25% of species were found in less than 1% of reads (Figure 4).

**Figure 5:**
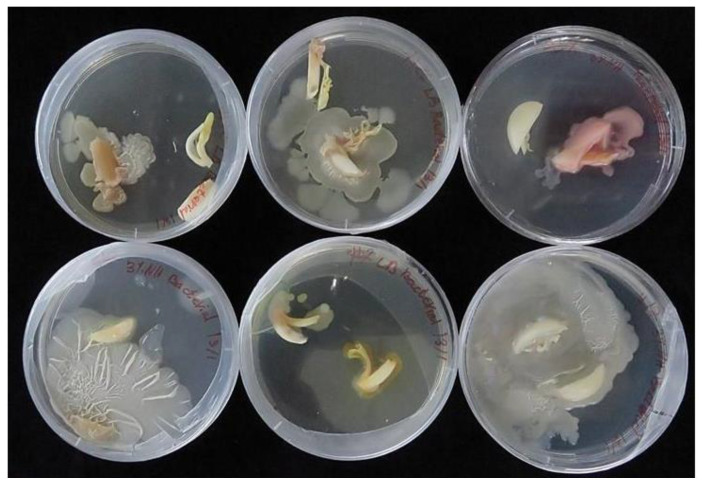
Representative samples of bacteria in garlic ‘Shani’. The bulbs were cultivated in an experimental field in Israel and subsequently stored for eight months in an open shed. Visual identification revealed a strong predominance of Bacillus.

We next estimated the culturable bacterial microbiome by isolating bacteria from garlic bulbs stored at 4 °C and inoculation on LB media (a non-selective media for bacteria) and MS medium with 3% sucrose, which is typically used for garlic tissue culture. The MS was used to estimate the presence of potential bacterial contaminants that can utilize the media, and therefore, may have a destructive effect on garlic tissue culture. We observed bacteria on 78% of samples on LB medium and 34% on MS medium, with surprisingly low variability, mostly Bacillus spp. and Pseudomonas spp. of the fluorescence group (Figure 5).

### 2.3. Virome of Garlic

**Figure 6:**
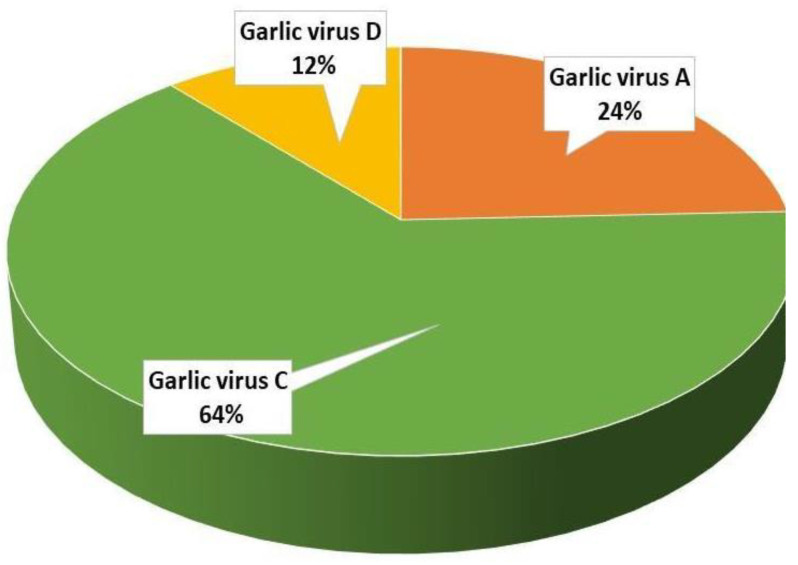
Virome complex associated with the transcriptome of garlic ‘Shani’. Contigs homologous to viruses were isolated, merged for isoforms of similar proteins, filtered for identification degree (above 80%), and compared with NCBI database.

**Figure 7:**
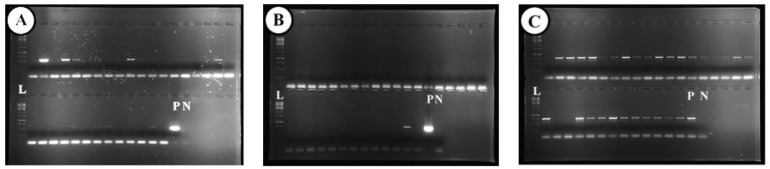
Virus detection in ‘Shani’ cloves using RT-PCR. The bulbs were cultivated in an experimental field in Israel and subsequently stored for eight months in an open shed. (**A**) Leek yellow strip virus (LYSV); (**B**) Onion yellow dwarf virus (OYDV), (**C**) Allexiviruses. L—Ladder, P—Positive control, N—Negative control.

Global analysis of transcriptome-associated viruses shows a predominant presence of the Allexiviruses GVA, GVC, and GVD, but no Poty- or Carla viruses (Figure 6). PCR analysis for the presence of a viral complex in the stored garlic bulbs identified Allexivirus in 94%, LYSV in 47% and OYDV in 6% of the samples (Figure 7).

### 2.4. In Vitro Sanitation and Pathogen Eradication

**Figure 8:**
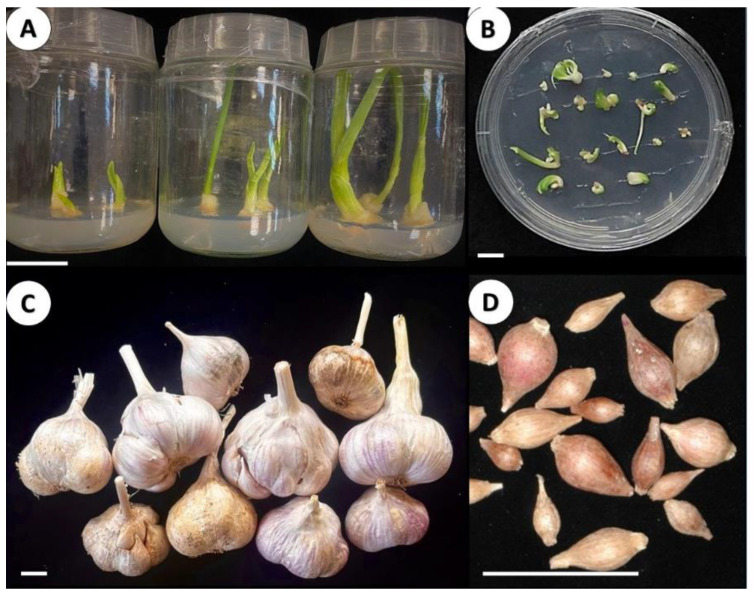
In vitro cultivation of garlic ‘Shani’. Scale = 2 cm (**A**) Isolated culture of shoot tips after seven, 11, and 14 days of regeneration. Fast formation of green leaves and roots is visible; (**B**) Regeneration of garlic explants 26 days after cryotherapy and cultivation in Petri dishes. Only several regenerants develop small leaf primordia; (**C**) Dormant bulbs produced after hardening and acclimation of shoot tip regenerants grown ex vitro for 6 months; (**D**) Dormant bulbs produced after hardening and acclimation of cryotherapy regenerants grown ex vitro for 16 months.

More than 80% of shoot tips of ‘Shani’ cultivated in vitro produced leaves 3–7 cm in length during two weeks of cultivation, and most of them formed roots without any additional treatment (Figure 8A). After two months of cultivation in vitro, these plantlets were ready for ex vitro hardening. At the end of the first hardening cycle, 90% of the ex vitro regenerants produced relatively large bulbs and entered dormancy after 6 months of ex vitro cultivation (Figure 8C).

On the other hand, cryotherapy resulted in a much lower survival rate of garlic explants (60%) and slower development of regenerants (Figure 8B). Only 20% of the regenerants were ready for hardening after six months of in vitro cultivation. Hardening, acclimation and growth for 10–16 months resulted in small dormant bulbs 0.5 cm in diameter (Figure 8D).

Shoot tip culture completely eradicated fungal infection, while in some cryotherapy regenerants fungi, mostly Penicillium, were identified, probably as a secondary infection after regeneration and cultivation period.

We next assessed the efficiency of cryotherapy in the elimination of endophytic bacteria. To do so, treated and untreated explants were surface sterilized, plated on LB media, and monitored for the appearance of bacteria after seven days. We observed a reduction in the presence of bacteria in the post-cryotherapy explants. Without cryotherapy treatment, bacteria appeared in 60% of explants, while after cryotherapy treatment bacteria appeared in 40%. In order to examine whether cryotherapy selectively affected the distribution of bacterial genera in explants, we characterized the bacterial isolates morphologically and identified the isolates by amplifying and sequencing the 16S rDNA (Table S1). The diversity of culturable bacteria in garlic explants was extremely low and was composed of Bacillus sp. and Pseudomonas sp. Interestingly, *P. gessardii* was repeatedly isolated from samples post cryotherapy treatment but was not found in the untreated explants.

**Figure 9:**
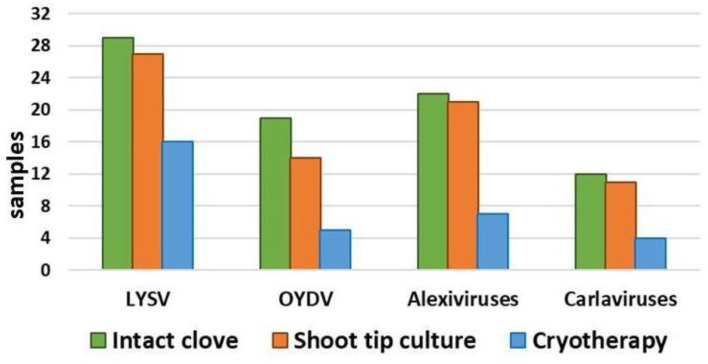
Comparison of viral infection in the population of intact cloves from 32 bulbs of garlic ‘Shani’ and regenerants after in vitro shoot tip culture and cryotherapy. LYSV—Leek yellow stripe virus; OYDV—Onion yellow dwarf virus.

Virus detection in the post-sanitation samples in comparison with intact garlic cloves showed higher efficiency of cryotherapy in comparison with shoot tip culture (Figure 9, Table S2). Cryotherapy completely eliminated LYSV and OYDV in 50 and 70% of samples, respectively. Still, although viral infection decreased in the post-cryo regenerant population, some regenerants remained infected. Shoot-tip culture practically did not eradicate viral infection (Figure 9).

## 3. Discussion

### 3.1. Garlic Bulb as a Holobiont

The garlic bulb represents an assemblage of plant hosts with numerous species of fungi, bacteria, and viruses that might make significant contributions to the plant’s life and contribute to agriculture sustainability [32].

However, the assessment of microbiomes associated with garlic tissues is challenging. Since garlic possesses high antibiotic activities due to the presence of active S-compounds, e.g., allicin [33,34], the routine biological assay of microflora using crushed tissue is not efficient. Upon tissue damage, allicin is produced from the amino acid alliin (S-allylcysteine sulfoxide) in a reaction that is catalyzed by the enzyme alliinase and can inhibit the proliferation of both bacteria and fungi [35]. In intact tissue, cell compartmentalization of alliin and alliinase restricts internal allicin production, and therefore, the symbiotic viruses, bacteria and fungi can survive in the internal tissues.

The metatranscriptomic approach used in this study resulted in the identification of numerous endophyte taxa in garlic tissues. This method is a cost-effective option to capture the biodiversity and abundance of samples [30]. We also enriched transcriptomic data by using culture methods, identification of bacteria by 16S rRNA gene sequence analysis, and PCR. It is still possible, however, that only microorganisms with higher resistance to allicin were able to survive sample preparation [36], so we may not have seen a complete representation of the microorganisms present.

Fungi are the most abundant components of the garlic holobiont. Metatranscriptome analysis reveals a large array of saprophytic and pathogenic fungal genera associated with plant tissues (Figure 1). Some have potentially deleterious effects, whereas others might be beneficial due to complex interactions with the plant, including the promotion of plant growth, inducing resistance and immunity, and assisting in the assimilation and translocation of nutrients [37]. The interaction of most of them (e.g., Dacryopinax which is found mainly in warm regions and has mycorrhizal association), [38], with the garlic plant is not clear yet. Others are known as devastating pathogens (Aspergillus and Fusarium), [39]. Interestingly, data have indicated the presence of two fungi with acaropathogenic abilities [40,41]. These fungi may induce plant immunity, growth promotion and suppression of the bulb mite *Rhizoglyphus robini* [42].

The bacterial microflora associated with garlic bulbs is also highly variable, with a predominance of Enterococcus (Figure 4). Although Enterococci are found mainly in animals, their presence on the plant surface has also been described [43]. A number of Enterococcus strains produce antimicrobial compounds including bacteriocins that are considered probiotics [44,45]. Antibiotic-resistant strains of enterococci have been found in animal hosts, plants, soil and water, and some possess novel resistance mechanisms [46]. The interplay of this group of bacteria with garlic needs further investigation to understand whether they are resistant to garlic S-compounds and their antibiotic traits.

Other endophytic bacteria (Brachybacterium, Cellulosimicrobium, and Bacillus) are known for plant growth promotion and antifungal activity [47]. In garlic, 2% of the bacterial pool was mapped to four Bacillus species. Symbiotic Bacillus spp. are of special interest since they improve plant response to pathogen attacks by triggering induced systemic resistance [48]. Thus, root-associated *B. saurashtrense* promotes the growth of Salicornia [49] and modulates physiological activity and abiotic stress in peanuts [50]. In garlic, Bacillus isolates have already been applied as biocontrol agents of Fusarium clove rot [51]. Inoculation of corn with Bacillus from the garlic rhizosphere promoted corn growth and yields and also inhibited in vitro development of the devastating parasite *Sclerotium cepivorum* [52]. Similarly, the inoculation of garlic meristems with Enterobacter and Burkholderia promoted the growth and physiological traits of garlic [53]. On the other hand, pathogenic Enterobacter caused decay in garlic production [54], and Pseudomonas species triggered garlic rot [55,56]. Therefore, bacterial endophytes include both negative and beneficial agents and their interaction with living garlic tissue is not clear yet.

Plant viruses are transmitted both horizontally and vertically, and similar to other vegetatively propagated crops (e.g., potato) [57], garlic viruses are spread within and between plant populations by aphids and thrips and during clonal propagation. Our recent studies have shown that garlic viruses are also transmitted by true seeds from infected mother plants and that some of them might even be integrated into the garlic genome [58]. Potyviruses are regarded as pathogenic agents in garlic [13], but our analysis of transcriptome-associated viruses shows a predominant presence of the Allexiviruses, while Poty- or Carla viruses were identified only by PCR analysis (Figure 6 and Figure 7). Favorable environmental conditions and proper production techniques can diminish the appearance and spread of viruses in commercial fields [59]. It is, therefore, possible that garlic plants can better handle a viral infection when other pathogens are being controlled.

### 3.2. Complete Elimination of Microflora by In Vitro Sanitation Is Not Essential for Garlic Production

Ridding plants of infections is important for food crop production and consumption, and a variety of agronomic and biotechnological methods have been developed for this purpose. However, the contradictions between the holobiont approach and the practical needs of pathogen eradication exist, especially in vegetatively propagated crops, such as potatoes or bananas [3]. In garlic, it is commonly accepted that healthy plant material and higher productivity require complete virus eradication [60,61]. However, biotechnological means are rarely used in commercial practice because the procedures are expensive, the survival rate of the virus-free propagules is low, and in-vitro multiplication is challenging [13,62]. 

Numerous publications are dedicated to in vitro sanitation as an efficient method of virus eradication in garlic, but other components of the microbiome were neglected in these studies. We employed two methods for in vitro sanitation—simple shoot tip culture and the more complicated and expensive cryotherapy. Both methods did not eradicate the virus load completely, but the number of infected regenerants was significantly lower after cryotherapy in comparison with shoot tip culture. In addition, our sanitation treatments reduced the bacterial load in the regenerants, but Bacillus and Pseudomonas spp. were still found in the tissues (Supplementary Table S1). Bacterial endophytes play both negative and positive roles in plant-pathogen interactions and their eradication may result in the decline of plant immune systems.

We found that the garlic mycobiome is located mainly in the external clove tissues (Table 1). Shoot-tip culture eradicated fungal flora from garlic regenerants, and can, therefore, be used as a sanitation procedure against fungal infections. When we employed shoot tip culture, we eliminated mainly pathogenic fungi from the external tissues. This process will make the garlic plant stronger with the ability to withstand other pathogens.

Taken together, shoot tip culture was not effective in virus elimination, but reduced bacterial load and eliminated fungal infections. This method resulted in fast explant regeneration and large and healthy bulbs in the first production cycle (Figure 8). On the other hand, cryotherapy is efficient in virus eradication but also kills other components of the garlic microbiome. Garlic plants sanitized by cryotherapy had a low survival rate, and a longer in vitro regeneration period [15,63] (Figure 8). Similarly, a large screening of the European garlic collections showed that virus elimination in vitro correlated with meristem size: 29% of viruses were eliminated in small meristem size compared to 8% for the larger size. The regeneration from the meristems was the opposite: 16% vs. 90% from small and large meristems, respectively [64].

**Figure 10:**
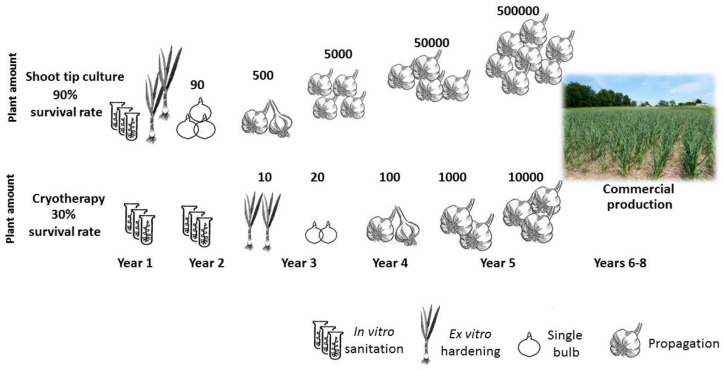
A trade-off between two pathways for pathogen eradication in garlic. Shoot tip culture is a fast and effective method, but it only partially reduces the pathogen load. On the other hand, cryotherapy is a powerful tool for pathogen eradication but is less efficient commercially.

From a commercial perspective, shoot tip culture might result in ca. 500,000 propagules from 100 explants in five years (Figure 10). Using cryotherapy, only about 10,000 propagules would be obtained by the end of five seasons from the initial 100 explants (Figure 10). Although post-cryotherapy regenerants could be virus-free, after a few years of commercial production they would be re-infected anyway and garlic stocks would need to be replaced by new sanitized propagation material. Therefore, even if garlic regenerants from shoot tip culture are not “totally free” of pathogens, they might provide a more efficient source for garlic production (Figure 10).

In conclusion, producing robust and valuable crops in sustainable systems without employing strong interventions to the plant’s biology is one of the main challenges of modern horticulture. Complete eradication in vitro eliminates both pathogenic and beneficial microbiota but does not guarantee immunity against future infections. Therefore, fast sanitation of garlic plant materials using the cultivation of shoot tips in vitro might provide an efficient alternative to more expensive techniques. The focus of garlic disease control should shift from the complete eradication of viruses to the study of the naturally occurring microbiome and identifying possible tools to protect the beneficial phytobiome from destruction.

## 4. Materials and Methods

### 4.1. Plant Material

Bulbs of garlic (*Allium sativum* L.) cv ‘Shani’ were used in this study in 2020–2022. Plant material was grown at Avnei Eitan Research Station in Northern Israel and in net-houses of the Agricultural Research Organization (ARO), The Volcani Institute, Rishon LeZion, Israel. Cloves were planted in November and bulbs were harvested in May. Common agriculture practices were applied during the growing season. In ARO plants were grown in a 30% shaded insect-proof net-house. Cloves were planted in 40 L plastic containers (40 cloves/container). The growing medium consisted of 50% ground coconut husk: 20% volcanic tuff particles: 20% peat: 10% compost (Even Ari, Israel). Irrigation was augmented with “Shefer” liquid fertilizer (N:P:K = 59:35:94 g/L, Dshanim, Israel). After harvest in May, bulbs were cleaned and stored in an open warehouse in ARO.

For sanitation experiments, bulbs harvested in May 2021 were stored either in an open warehouse or at 4 °C. After eight months in storage, 32 bulbs were numbered and separated into cloves. From each bulb, we tested cloves for (1) fungal infection in four cloves; (2) bacteria presence in two cloves; (3) PCR detection of Onion yellow dwarf virus (OYDV), Leek yellow stripe virus (LYSV), Carlaviruses and Allexiviruses in one clove. The remaining cloves were divided between sanitation by cryotherapy and shoot tips in vitro.

### 4.2. Garlic Microbiome

#### 4.2.1. Transcriptome Analysis

To assess a large number of in-plant endophytes, we used a culture-independent approach and analyzed the full transcriptome catalog of garlic bulb tissues [65]. The bulbs of the garlic cultivar ‘Shani’ were produced in a commercial field in Israel, using regular growth practices. Internal buds and storage leaves were sampled in three replicates, 10 cloves from different bulbs in each replicate, pooled together and dipped immediately in liquid nitrogen, thereafter stored at −80 °C until RNA isolation.

For RNA isolation and sequencing procedures, total RNA was extracted according to the CTAB protocol [66]. Sample purity and integrity were verified by RNA 6000 Nano Assay with an Agilent 2100 BioAnalyzer (Agilent Technologies, Waldbronn, Germany) with a minimum RNA integrated number value of 7, and then samples were treated with DNase (Epicenter, Madison, WI, USA) according to the supplier’s instructions. Total RNA samples were shipped to the Roy J. Carver Biotechnology Center, W.M. Keck Center for Comparative and Functional Genomics, Urbana, IL, USA, for library preparation and sequencing. Twelve libraries of 100-nucleotide-long single-ended RNA sequences were constructed and used for transcriptome sequencing using Illumina HiSeq 2000 Illumina Inc, SanDiego, CA and TrueSeq protocols. The RNA-Seq data were deposited in the NCBI sequence read archive (SRA) as bioproject PRJNA384121 and biosample SAMN06828997.

A total of 122.9 million cleaned reads, obtained after processing and cleaning, were assembled de novo using Trinity software (v2.9.1) [67]. The assembled transcriptome was used for a search against the NCBI non-redundant (nr) protein database, using DIAMOND v.3 software [68]. The results were exported to Blast2GO version 4.0 [69] for taxonomy assignments. To filter the transcriptome assembly, a set of criteria were applied: (1) extracting contig homology to bacteria, fungi, or viruses; (2) merging contigs of isoforms of very similar proteins; (3) selecting isoforms above 80% identification degree, (4) comparing contigs with known data on NCBI database. Clean reads were mapped on the bacteria, fungi, and virus contigs using the BWA-MEM algorithm [70]. SOAP coverage (version 2.7.7) was used for depth estimation of each contig [71]. The list of viruses, bacteria, and fungi was verified by the APS list of Diseases of Onion and Garlic [72].

The top 10 results from bacteria and fungi lists generated in our transcriptome analysis were chosen for comparison in the samples before and after in vitro sanitation procedures.

#### 4.2.2. Assessment of Fungal Flora

Mycoflora were assessed separately for the external and internal clove tissue of 32 bulbs. Cloves were peeled to remove the dry external scales and rinsed under running water for one minute. For the external tissue sampling, the bottom part of a basal plate was cut into thin discs. To assess the infection of the inner tissues, cloves were washed with soap and dipped in 70% ethanol for one minute. Under sterile conditions, the material was then submerged for 20 min in 3% sodium hypochlorite solution with 0.01% *v/v* Tween 20, rinsed with sterile distilled water, and cut into slices of the basal plate and the lower parts of the leaf primordia. Four to six slices were placed in 90 mm Petri dishes containing potato dextrose agar (PDA, 39 gr/L) and 1µg/mL chloramphenicol (Sigma, Merck KGaA, Darmstadt, Germany), pH 5.6, and kept in the dark at 25 ± 1 °C for seven days until fungal colonies were evident [41]. Then, a piece of mycelia from each colony was aseptically transferred to a fresh culture media for further fungal propagation and identification. Hyphae-bearing conidiophores were sampled from each isolate for morphological characterization by light microscopy as per Lacey [73].

#### 4.2.3. Assessment of Bacterial Flora

The routine procedure for plant sample preparation includes incubation of crushed tissues for seven days at 27 °C. However, we failed to culture any bacteria from garlic tissue following this procedure. Therefore, surface-sterilized cloves were cut vertically into quarters and placed on Petri dishes containing two different media: 15 mL Luria-Bertani (LB, 20 g/L) and Cycloheximide (1 mL/L) [74], or 15 mL of 4.4 g/L MS [75], agar (9.5 g/L), sucrose (30 g/L), pH 6. Plates were kept at 27 °C in the dark, for seven days.

For identification of bacteria by 16S rRNA gene sequence analysis, bacterial isolation spreads of visually different colonies were cultivated on fresh media for 24–48 h. Isolates were diluted in double distilled water and heated to 95 °C for five minutes. 1 µL of diluted samples was used as templates for PCR, mixed with 12.5 µL Q5^®^ High-Fidelity 2X Master Mix chemical, not equipment, 1.25 µL Uni16S—U1492R (GGT TAC CTT GTT ACG ACT T) and Nuclease-Free Water to 25 µL total. The amplification protocol consisted of three minutes at 94 °C, then 30 cycles of amplification as follows: denaturation at 94 °C for 30 s, annealing at 50 °C for 30 s, and extension at 72 °C for 120 s. The PCR product was loaded on an agarose gel composed of Agarose 1% (CLS-AG500) Cleaver Scientific Ltd. Rugby, UK diluted in an electrophoresis buffer Tris Acetate-EDTA (TAE) (Biological Industries, Israel). Amplicons were purified using Gel/PCR DNA Fragment Extraction Kit (Geneaid, Taiwan) and sequenced by Sanger technology (Hylabs, Rehovot, Israel). Identification of bacterial sequences was conducted by using National Center for Biotechnology Information (NCBI, https://blast.ncbi.nlm.nih.gov/Blast.cgi?PROGRAM=blastn&PAGE_TYPE=BlastSearch&LINK_LOC=blasthome (accessed on 1 April 2023)).

#### 4.2.4. PCR Detection of Poty-, Allexi- and Carlaviruses

RNA extraction was performed using the AccuPrep viral RNA Extraction Kit (Bioneer Corporation, Daejeon, Republic of Korea), according to the manufacturer’s instructions. For cDNA synthesis, the RevertAid Reverse Transcriptase cDNA Synthesis Kit was used according to the manufacturer’s instructions (Thermo Fisher Scientific, Waltham, MA, USA). cDNA samples were kept at 4 °C before the PCR reaction.

For LYSV and OYDV amplification, the relevant viral segments were obtained by enzymatic reaction of Hy-taq ready mix (×2) 10 µL added with Forward 9147 or Forward 9565 primer 1 µL, DDW 7 µL, and a Reverse primer RS1 1 µles and added to 1 µL of sampled cDNA. Specific universal primer RS1 enables the isolation of Potyviral conserved genomic sequences, including LYSV and OYDV. The primers were selected for the demarcation of specific segments (Table 2).

Allexivirus amplification was conducted by enzymatic reaction of Hy-taq ready mix (×2) 10 µL added with Forward 7457 primer 1 µL, DDW 7 µL and Reverse primer 8182, added to 1 µL of sampled cDNA. Degenerative primers were used to amplify all Allexivirus strands.

Carlavirus amplification was conducted by enzymatic reaction of Hy-taq ready mix (×2) 10 µL added with Forward primer 5296 1 µL, DDW 7 µL and Reverse primer 6246, added to 1 µL of sampled cDNA. Carlavirus primers were used to amplify Garlic latent and Garlic common virus.

The reaction mix and samples were subjected to 94 °C for five minutes. For Potyviruses, the reaction mixture was subjected to 38 cycles of amplification as follows: denaturation at 94 °C for 30 s, annealing at 60 °C for 30 s, and extension at 72 °C for 60 s. For Allexi- and Carlaviruses the amplification protocol consisted of 40 cycles of amplification as follows: denaturation at 94 °C for 30 s, annealing at 60 °C for 30 s, and extension at 72 °C for 60 s, to end the annealing process. The PCR product was loaded on an agarose gel composed of Agarose 1.7% (CLS-AG500) Cleaver Scientific Ltd. and diluted in TAE (Biological Industries, Beit Haemek Israel), 1 kb. DNA ladder (Thermo Fisher Scientific, Walthma, MA, USA) was used as a size scale [76]. Two controls were used, (1) negative—without cDNA but with an addition of DDW 1 µL, and (2) positive—viral plasmids produced by [D. Gelbart,2020, pers. comm.].

### 4.3. Sanitation Procedures In Vitro

#### 4.3.1. Cryotherapy

The procedures include cutting the clove under sterile conditions to excise a 1 mm^2^ explant of the apical meristem [15,77].

In the preliminary experiment, we optimized the conditions of tissue vitrification prior to submerging in liquid nitrogen. For that, four groups of 40 explants each were treated with Plant Vitrification Solution PVS3 before submerging to liquid nitrogen as follows: (1) PVS3 50% *w/v* sucrose and 50% *w/v* glycerol solution, sterilized for 15 min in the autoclave (121 °C); (2) PVS3 50% *w/v* sucrose and 50% *w/v* glycerol solution, sterilized by syringe filter (PVDF membrane, 0.45-micrometer pore size); (3) PVS3 40% *w/v* sucrose and 40% *w/v* glycerol solution, sterilized by syringe filter; (4) Control group was treated by filtered PVS3 50% *w/v* sucrose and 50% *w/v* glycerol, but without submerge to liquid nitrogen.

Number of regenerants was counted two weeks after cryotherapy. The survival rate of the explants after cryotherapy with 50% (*w/v*) sucrose and 50% (*w/v*) glycerol, serialized by 15 min autoclave or syringe filter was 20–25%). At the same time, a diluted version of PVS3 in concentration of 40% (*w/v*) sucrose and 40% (*w/v*) glycerol and filter sterilization resulted in a survival rate of 60%, similar to that of the explants exposed to the full protocol but without cryo-procedures in LN (control). Therefore, PVS3 in 40% (*w/v*) sucrose and 40% (*w/v*) glycerol concentration sterilized by syringe filter was chosen to be for ‘Shani’ cryotherapy.

In the main experiments, the explants from the apical meristems were placed in Petri dishes with 4.4 g/L MS medium supplemented with sucrose 100 g/L, agar 9.5 g/L, Indole-3-acetic acid (IAA) 100 µL/L, 6- (γ, γ-Dimethylallylamino) Purine (2iP) 500 µL/L, pH 6. The plates were kept in a dark room at 20 °C for 24 h. The next day the explants were moved to 2 mL cryotubes, 10 explants each. One ml of sterile loading solution (0.4 M sucrose + 2M glycerol + 4.4 g/L MS, with pH set to 5.8) was added to the cryotubes for 20 min. Then the loading solution was replaced by 1 mL of sterile PVS3 40% (0.58 M sucrose + 2.16 M glycerol + 4.4 g/L MS) sterilized by syringe filter (PVDF membrane, 0.45-micrometer pore size).

Cryotubes were slightly shaken and allowed to rest at room temperature for 2 h. The PVS3 was replaced by fresh 0.5 mL PVS3 and the cryotubes were submerged in liquid nitrogen for one hour, and then placed in a 40 °C water bath for two minutes. The defrosted PVS3 was removed and 1 mL of sterile sucrose medium (1.2 M sucrose + 4.4 g/L MS, pH 5.8) was added for 10 min. Then the medium was removed and explants were placed on Petri dishes containing 4.4 g/L MS medium supplemented with sucrose 30 g/L, agar 9.5 g/L, IAA 100 µL/L, 2iP 500 µL/L, pH 5.8. Plates were kept in the dark at 20 °C for seven days before moving to a 10/14 light cycle, under fluorescent lamp G13 36 W 6400 K, light intensity 2700 mL. this is standartd lamps, we purchase them on-line

#### 4.3.2. Shoot Tip Culture

Shoot tips of 1–1.5 mm^2^ were excised from surface-sterilized cloves, introduced into ventilated magenta boxes with 40 mL MS medium 4.4 g/L supplemented with sucrose 30 g/L, agar 9.5 g/L, IAA 100 µL/L, 2iP 500 µL/L, pH 6, and grown at 20 °C and 10/14 h light/dark cycle, under fluorescent lamp G13 36 W 6400 K, light intensity 2700 mL.

#### 4.3.3. Post-Culture Procedures No Conclusion Section in This Paper

After 40 days of regeneration after cryotherapy or shoot tip culture, segments of the new leaves were sampled for pathogen identification.

For hardening, the regenerants were transplanted into tray cells with potting mixture and grown in the chambers at 20 °C and 80–90% humidity [20]. Plantlets have been acclimatized for 2 weeks and then transplanted into 1 L pots with planting mixture (50% ground coconut husk: 20% volcanic tuff particles: 20% peat: 10% compost (Even Ari, Beit Elazari Israel) and grown in an insect-proof net house in ARO the Volcani Center.

## Figures and Tables

**Table 1:** Percentage of fungi in the external and internal sections of garlic ‘Shani’ cloves.

Fungal Genus	Warehouse, Ambient Conditions		Storage at Dark, 4 °C for 8 Months	
	Internal	External	Internal	External
Penicillium	13%	60%	19%	84%
Aspergillus	0%	53%	6%	34%
Fusarium	0%	20%	3%	38%
Mucor	0%	0%	0%	3%
No fungi	87%	13%	72%	3%

**Table 2:** Primers used in the RT-PCR. Source: NCBI (National Center for Biotechnology Information) and D. Gelbart, unpublished, 2014.

Target Virus	Primer	Sequence (5′–3′)	Expected Fragment Size, bp
RS1 Conserved Potyvirus sequence	9725R	5′- TGC TGT GTG CCT CTC CGT GTC CTC -3′	
LYSV	Forward 9147	5′- GAG GAA AGT CAA TAC TTA AC-3′	578
OYDV	Forward 9565	5′- GAG GAT GCA CAA TCA AG - 3’	714
Degenerative Allexiviruses	Forward 7457	5′- GCW TGG RCB TGC TAY CAC AAY GG -3′	725
	Reverse 8182	5′- CYT TCA GCA TRT AGC TTA GCR GGT CC - -3′	
Carlaviruses	Forward 5296	5′- CTG AAT CAG ATT ATG AAG CTT TTG ATG C- 3’	949
	Reverse 6246	5’ - CAA TCA CCC AGC TGG TAT TCG TC - 3’	

## Data Availability

Data are presented within the article and Supplementary Materials.

## Supplementary Materials

The following supporting information can be downloaded at: https://www.mdpi.com/article/10.3390/plants12244125/s1, Table S1: Culturable bacteria isolated from garlic shoot tip after cryotherapy treatment; Table S2: PCR detection of Potyviruses LYSV and OYDV, Allexiviruses and Carlaviruses in garlic tissues prior to in vitro culture, and following sanitation via shoot tip culture and cryotherapy. 32 garlic bulbs was separated into cloves and each genotype/clove served as an explant in different in vitro experiments. + detected; - not detected.
