# Supplementary material for: Pathogen Eradication in Garlic in the Phytobiome Context: Should We Aim for Complete Cleaning?
Source: Plants (Basel). 2023 Dec 10;12(24):4125. doi: 10.3390/plants12244125 (PMC10747685; doi:10.3390/plants12244125)
Supplement: Supplementary file 1 [file plants-12-04125-s001.zip › Table S1.pdf]

Table S1. Culturable bacteria isolated from garlic shoot tip after cryotherapy treatment

**Isolate number**	**Cryotherapy**	**Closest homolog**	**Identity to closest homolog**	**Closest homolog locus tag**
111	Non-treated explants	*Bacillus subtilis* subsp. *subtilis* strain WXZS3	98%	OL468538.1
112	Non-treated explants	*Bacillus subtilis* sp.	99%	MT111029.1
113	Non-treated explants	*Bacillus subtilis* subsp. *subtilis* strain QSYS10	98%	OL468493.1
120	Non-treated explants	*Bacillus subtilis* subsp. *subtilis* strain QSYS5	99%	OL468505.1
114	Non-treated explants	*Pseudomonas lactis* strain Boxwood-SP	91%	MK423985.1
121	Treated explants	*Bacillus subtilis* subsp. *subtilis* strain WXZP7	99%	OL468530.1
116	Treated explants	*Pseudomonas gessardii* strain OBE3	99%	MN685265.1
117	Treated explants	*Pseudomonas gessardii* strain OBE3	99%	MN685265.1
118	Treated explants	*Pseudomonas gessardii* strain OBE3	98%	MN685265.1
119	Treated explants	*Pseudomonas gessardii* strain OBE3 16S	98%	MN685265.1
122	Treated explants	*Pseudomonas gessardii* strain OBE3 16S	98%	MN685265.1
