# Supplementary material for: Pathogen Eradication in Garlic in the Phytobiome Context: Should We Aim for Complete Cleaning?
Source: Plants (Basel). 2023 Dec 10;12(24):4125. doi: 10.3390/plants12244125 (PMC10747685; doi:10.3390/plants12244125)
Supplement: Supplementary file 1 [file plants-12-04125-s001.zip › Table S2.pdf]

Table S2. PCR detection of Potyviruses LYSV and OYDV, Allexiviruses and Carlaviruses in garlic tissues prior to *in vitro* culture, and following sanitation via shoot tip culture and cryotherapy. 32 garlic bulbs was separated into cloves and each genotype/clove served as an explant in different *in vitro* experiments. + detected; - not detected

**Sample number**	**LYSV**			**OYDV**			**Allexiviruses**			**Carlaviruses**		
	**Cloves no treatment**	**Shoot tip**	**Cryotherapy**	**Cloves no treatment**	**Shoot tip**	**Cryotherapy**	**Cloves no treatment**	**Shoot tip**	**Cryotherapy**	**Cloves no treatment**	**Shoot tip**	**Cryotherapy**
**1**	+	+	+	-	-	-	+	-	-	-	-	+
**2**	+	+	+	-	-	-	+	+	-	-	-	-
**3**	+	-	-	-	-	-	+	-	-	-	-	-
**4**	+	-	-	-	+	-	+	-	-	-	-	-
**5**	+	+	+	-	-	-	+	+	+	-	-	-
**6**	+	+	+	+	+	+	-	+	-	-	+	-
**7**	+	+	-	-	+	-	-	+	-	-	+	-
**8**	+	+	+	+	+	+	+	-	-	+	+	-
**9**	-	-	-	+	+	-	-	-	+	+	+	-
**10**	+	+	-	+	+	-	+	+	-	-	-	-
**11**	+	+	-	-	+	-	+	-	+	-	-	-
**12**	+	+	-	+	+	-	+	+	-	+	-	-
**13**	+	-	+	+	+	+	+	+	+	+	-	-
**14**	+	+	+	-	-	-	+	+	+	-	-	-
**15**	+	+	-	+	+	-	+	+	+	+	+	-
**16**	+	+	+	+	+	-	-	-	-	-	-	-
**17**	+	+	+	-	-	-	-	-	-	+	-	-
**18**	+	+	-	+	-	-	+	+	-	-	-	-
**19**	+	+	+	-	-	-	-	-	-	-	-	-
**20**	+	+	-	+	+	-	+	+	-	+	-	-
**21**	+	+	-	-	-	-	-	+	-	-	-	-
**22**	+	+	+	-	-	-	+	+	-	-	-	-
**23**	+	+	+	+	-	-	+	+	-	+	+	+
**24**	+	+	-	+	-	-	+	+	-	+	+	-
**25**	-	+	-	+	-	-	+	-	-	-	-	-
**26**	+	+	+	+	-	-	+	+	-	+	+	-
**27**	+	+	-	+	-	-	-	+	-	+	+	-
**28**	+	-	-	+	-	-	+	+	-	-	+	-
**29**	-	+	+	+	-	-	+	+	-	-	-	-
**30**	+	+	-	-	-	-	+	+	-	-	-	-
**31**	+	+	+	+	+	+	-	+	+	-	-	+
**32**	+	+	+	+	+	+	-	-	-	+	+	+
